# Weak forms of continuity in *I*-double gradation fuzzy topological spaces

**DOI:** 10.1186/2193-1801-1-19

**Published:** 2012-09-25

**Authors:** A Ghareeb

**Affiliations:** 1 Mathematics Department, Faculty of Science, South Valley University, Qena, Egypt; 2 Department of Mathematics, College of Science in Al-Zulfi, Majmaah University, Al-Zulfi, Kingdom of Saudi Arabia

## Abstract

In this paper, we introduce and characterize double fuzzy weakly preopen and double fuzzy weakly preclosed functions between *I*-double gradation fuzzy topological spaces and also study these functions in relation to some other types of already known functions.

## Introduction

In the history of science, new theories have always been necessary in order for existing scientific theories to progress and this will continue to be true in the future. Two examples of essentially different mathematical theories that deal with the concept of uncertainty are probability theory and the theory of fuzzy sets. Whereas probability theory has a history of around 360 years, the theory of fuzzy sets is little more than 50 years old. Since the 1960s fuzzy methods have entered the scientific and technological world, good theoretical progress (e.g., fuzzy logic, fuzzy probability theory, fuzzy topology, fuzzy algebra) has been made, and there have been technical advances in various areas (e.g., fuzzy control, fuzzy expert systems, fuzzy clustering and data mining).

Chang (1968); Lowen (1976); Šostak (1985); Kubiak (1985); Samanta and Mondal (1997,2002) and many others contributed a lot to the field of Fuzzy Topology. In recent years Fuzzy Topology has been found to be very useful in solving many practical problems. Shihong Du et. al. (2005) are currently working to fuzzify the 9-intersection Egenhofer model Egenhofer and Franzosa (1991); Herring and Egenhofer (1991) for describing topological relations in Geographic Information Systems (GIS) query. In El-Naschie (1998,2000), El-Naschie has shown that the notion of Fuzzy Topology is applicable to quantum particle physics and quantum gravity in connection with String Theory and *e*^*∞*^ Theory. Tang (2004) has used a slightly changed version of Chang’s fuzzy topological space to model spatial objects for GIS databases and Structured Query Language (SQL) for GIS.

In this paper, we will introduce the concepts of double fuzzy weakly preopen and double weakly preclosed functions in *I*-double gradation fuzzy topological spaces. Their properties and the relationships between these functions and other functions introduced previously are investigated.

## Preliminaries

Throughout this paper, let *X* be a nonempty set and *I* is the closed unit interval [0,1]. *I*_∘_=(0,1] and *I*_1_=[0,1). The family of all fuzzy subsets on *X* denoted by *I*^*X*^. By  and , we denote the smallest and the greatest fuzzy subsets on *X*. For a fuzzy subset *λ*∈*I*^*X*^,  denotes its complement. Given a function , *f*(*λ*) and *f*^−1^(*λ*) define the direct image and the inverse image of *f*, defined by  and *f*^−1^(*ν*)(*x*)=*ν*(*f*(*x*)), for each *λ*∈*I*^*X*^, *ν*∈*I*^*Y*^, and *x*∈*X*, respectively. For fuzzy subsets *λ* and *μ* in *X*, we write *λqμ* to mean that *λ* is quasi coincident (q-coincident) with *μ*, that is, there exists at least one point *x*∈*X*such that *λ*(*x*) + *μ*(*x*)>1. Negation of such a statement is denoted as . Notions and notations not described in this paper are standard and usual.

### Definition 2.1

[(*Samanta and Mondal* (1997,2002); *Garcia and Rodabaugh* (2005)] An *I*-double gradation fuzzy topology (*ττ*^∗^) on *X* is a pair of maps *τ*, *τ*^∗^:*I*^*X*^→*I*, which satisfies the following properties:

- (O1)  for each *λ*∈*I*^*X*^.
- (O2) *τ*(*λ*_1_∧*λ*_2_)≥*τ*(*λ*_1_)∧*τ*(*λ*_2_) and *τ*^∗^(*λ*_1_∧*λ*_2_)≤*τ*^∗^(*λ*_1_)∨*τ*^∗^(*λ*_2_) for each *λ*_1_, *λ*_2_∈*I*^*X*^.
- (O3)  and  for each *λ*_*i*_∈*I*^*X*^, *i*∈*Γ*.

The triplet (*X*,*τ*,*τ*^∗^) is called an *I*-double gradation fuzzy topological spaces (*I*-dfts, for short). A fuzzy set *λ* is called an (*r*,*s*)-fuzzy open ((*r*,*s*)-fo, for short) if *τ*(*λ*)≥*r* and *τ*^∗^(*λ*)≤*s*. A fuzzy set *λ* is called an (*r*,*s*)-fuzzy closed ((*r*,*s*)-fc, for short) set iff  is an (*r*,*s*)-fo set. Let  and  be two *I*-dfts’s. A function  is said to be a double fuzzy continuous iff *τ*_1_(*f*^−1^(*ν*))≥*τ*_2_(*ν*) and  for each *ν*∈*I*^*Y*^.

There was a question we must ask ourselve before starting to present our results, which was: *Is it useful to introduce new concepts to I-double gradation fuzzy topological spaces*?

We could know that double (initially, intuitionistic) fuzzy sets (and hence double fuzzy topological spaces) deal with ambiguity in a way better than fuzzy sets. In addition to that, double fuzzy topological spaces is a generalization of some other kinds of topological spaces; we can get fuzzy topological spaces in Chang’s sense , where 

Also, when the conditions *τ*^∗^(*λ*)=1−*τ*(*λ*) and *τ*(*λ*) + *τ*^∗^(*λ*)≮1 achieved in Definition 2.1, we get the definition of fuzzy topological spaces in Kubiak- Šostak’s sense Kubiak (1985); Šostak (1985). If we use 2^*X*^instead of *I*^*X*^, the resulting topological structure will be called double gradation fuzzifying topological spaces (A new structure mentioned for the first time in Bhaumik and Abbas 2008). Besides, we can also get the general topological spaces.

### Theorem 2.1

*[(Çoker and Demirci*^1996^*; Lee and Im (*2001*)]* Let (*Xττ*^∗^) be an *I*-dfts. Then for each *r*∈*I*_0_, *s*∈*I*_1_ and *λ*∈*I*^*X*^, we define an operator *C*_*τ*,*τ*_^∗^:*I*^*X*^×*I*_0_×*I*_1_→*I*^*X*^ as follows: 

For *λ*, *μ*∈*I*^*X*^, *r*_1_*r*_2_∈*I*_0_and *s*_1_*s*_2_∈*I*_1_, the operator *C*_*τ*,*τ*_^∗^satisfies the following statements:

- (C1) ,
- (C2) *λ*≤*C*_*τ*,*τ*_^∗^(*λ*,*r*,*s*),
- (C3) *C*_*τ*,*τ*_^∗^(*λ*,*r*,*s*)∨*C*_*τ*,*τ*_^∗^(*μ*,*r*,*s*)=*C*_*τ*,*τ*_^∗^(*λ*∨*μ*,*r*,*s*),
- (C4) *C*_*τ*,*τ*_^∗^(*λ*,*r*_1_,*s*_1_)≤*C*_*τ*,*τ*_^∗^(*λ*,*r*_2_,*s*_2_) if *r*_1_≤*r*_2_and *s*_1_≥*s*_2_,
- (C5) *C*_*τ*,*τ*_^∗^(*C*_*τ*,*τ*_^∗^(*λ*,*r*,*s*),*r*,*s*)=*C*_*τ*,*τ*_^∗^(*λ*,*r*,*s*).

### Theorem 2.2

*[(Çoker and Demirci*^1996^*; Lee and Im*^2001^*)]* Let (*Xττ*^∗^) be an *I*-dfts. Then for each *r*∈*I*_0_, *s*∈*I*_1_ and *λ*∈*I*^*X*^, we define an operator *I*_*τ*,*τ*_^∗^:*I*^*X*^×*I*_0_×*I*_1_→*I*^*X*^as follows: 

For *λμ*∈*I*^*X*^, *rr*_1_*r*_2_∈*I*_0_and *ss*_1_*s*_2_∈*I*_1_, the operator *I*_*τ*,*τ*_^∗^satisfies the following statements:

,
,
3. *I*_*τ*,*τ*_^∗^(*λ*,*r*,*s*)≤*λ*,
4. *I*_*τ*,*τ*_^∗^(*λ*,*r*,*s*)∧*I*_*τ*,*τ*_^∗^(*μ*,*r*,*s*)=*I*_*τ*,*τ*_^∗^(*λ*∧*μ*,*r*,*s*),
5. *I*_*τ*,*τ*_^∗^(*λ*,*r*_1_,*s*_1_)≥*I*_*τ*,*τ*_^∗^(*λ*,*r*_2_,*s*_2_) if *r*_1_≤*r*_2_and *s*_1_≥*s*_2_,
6. *I*_*τ*,*τ*_^∗^(*I*_*τ*,*τ*_^∗^(*λ*,*r*,*s*),*r*,*s*)=*I*_*τ*,*τ*_^∗^(*λ*,*r*,*s*),
7. If *I*_*τ*,*τ*_^∗^(*C*_*τ*,*τ*_^∗^(*λ*,*r*,*s*),*r*,*s*)=*λ*, then .

### Definition 2.2

Let (*X*,*τ*,*τ*^∗^) be an *I*-dfts. For *λ*∈*I*^*X*^, *r*∈*I*_0_and *s*∈*I*_1_.

(1) *λ* is called (*r*,*s*)-fuzzy preopen ((*r*,*s*)-fpo, for short) if *λ*≤*I*_*τ*,*τ*_^∗^(*C*_*τ*,*τ*_^∗^(*λ*,*r*,*s*),*r*,*s*). A fuzzy set *λ* is called (*r*,*s*)-fuzzy preclosed ((*r*,*s*)-fpc, for short) iff  is (*r*,*s*)-fpo set. The (*r*,*s*)-fuzzy preinterior of *λ*, denoted by *PI*_*τ*,*τ*_^∗^(*λ*,*r*,*s*) is defined by 

The (*r*,*s*)-fuzzy preclosure of *λ*, denoted by *PC*_*τ*,*τ*_^∗^(*λ*,*r*,*s*) is defined by 

(2) *λ* is called (*r*,*s*)-fuzzy regular open ((*r*,*s*)-fro, for short) if *λ*=*I*_*τ*,*τ*_^∗^(*C*_*τ*,*τ*_^∗^(*λ*,*r*,*s*),*r*,*s*). A fuzzy set *λ* is called (*r*,*s*)-fuzzy regular closed ((*r*,*s*)-frc, for short) iff  is (*r*,*s*)-fro set.

(3) *λ* is called (*r*,*s*)-fuzzy *α*-open ((*r*,*s*)-f*α*o, for short) if *λ*≤*I*_*τ*,*τ*_^∗^(*C*_*τ*,*τ*_^∗^(*I*_*τ*,*τ*_^∗^(*λ*,*r*,*s*),*r*,*s*),*r*,*s*). A fuzzy set*λ*is called (*r*,*s*)-fuzzy *α*-closed ((*r*,*s*)-f*α*c, for short) iff  is (*r*,*s*)-f*α*o set.

### Theorem 2.3

Let (*X*,*τ*,*τ*^∗^) be an *I*-dfts. For *λ*∈*I*^*X*^, *r*∈*I*_0_and *s*∈*I*_1_.

1. *λ*is (*r*,*s*)-fpo (resp. (*r*,*s*)-fpc) iff *λ*=*PI*_*τ*,*τ*_^∗^(*λ*,*r*,*s*) (resp. *λ*=*PC*_*τ*,*τ*_^∗^(*λ*,*r*,*s*)),
2. *I*_*τ*,*τ*_^∗^(*λ*,*r*,*s*)≤*PI*_*τ*,*τ*_^∗^(*λ*,*r*,*s*)≤*λ*≤*PC*_*τ*,*τ*_^∗^(*λ*,*r*,*s*)≤*C*_*τ*,*τ*_^∗^(*λ*,*r*,*s*),
and .

### Definition 2.3

Let  be a function from an *I*-dfts  into an *I*-dfts . The function *f* is called:

1. double fuzzy preclosed if *f*(*λ*) is (*r*,*s*)-fpc set in *I*^*Y*^for each *λ*∈*I*^*X*^, *r*∈*I*_0_and *s*∈*I*_1_; , ,
2. double fuzzy open if *τ*_2_(*f*(*λ*))≥*τ*_1_(*λ*) and  for each *λ*∈*I*^*X*^, *r*∈*I*_0_and *s*∈*I*_1_,
3. double fuzzy almost open if *τ*_2_(*f*(*λ*))≥*r*and  for each (*r*,*s*)-fro set *λ*∈*I*^*X*^, *r*∈*I*_0_and *s*∈*I*_1_.

### Definition 2.4

Let  be a function from an *I*-dfts  into an *I*-dfts . The function *f* is called:

1. double fuzzy weakly open if  for each *λ*∈*I*^*X*^, *r*∈*I*_0_and *s*∈*I*_1_; *τ*_1_(*λ*)≥*r*and ,
2. double fuzzy *α*-open if *f*(*λ*) is (*r*,*s*)-f*α*o in *I*^*Y*^for each *λ*∈*I*^*X*^, *r*∈*I*_0_and *s*∈*I*_1_; *τ*_1_(*λ*)≥*r*and .

### Definition 2.5

Let (*X*,*τ*,*τ*^∗^) be an *I*-dfts, *μ*∈*I*^*X*^, *x*_*t*_∈**P**(*X*), *r*∈*I*_0_and *s*∈*I*_1_where **P**(*X*) is the family of all fuzzy points in *X*. *μ*is called an (*r*,*s*)-fuzzy open Q-neighborhood of *x*_*t*_if *τ*(*μ*)≥*r*, *τ*^∗^(*μ*)≤*s*and *x*_*t*_*qμ*. We denote the set of all (*r*,*s*)-fuzzy open Q-neighborhood of *x*_*t*_by **Q**_*τ*,*τ*_^∗^(*x*_*t*_,*r*,*s*).

### Definition 2.6

Let (*X*,*τ*,*τ*^∗^) be an *I*-dfts, *λ*∈*I*^*X*^, *x*_*t*_∈**P**(*X*), *r*∈*I*_0_and *s*∈*I*_1_. *x*_*t*_is called (*r*,*s*)-fuzzy *θ*-cluster point of *λ*if for every *μ*∈**Q**_*τ*,*τ*_^∗^(*x*_*t*_,*r*,*s*), we have *C*_*τ*,*τ*_^∗^(*μ*,*r*,*s*)*qλ*. We denote . Where *D*_*τ*,*τ*_^∗^(*λ*,*r*,*s*) is called (*r*,*s*)-fuzzy *θ*-closure of *λ*.

### Theorem 2.4

Let (*X*,*τ*,*τ*^∗^) an *I*-dfts. For *λ*, *μ*∈*I*^*X*^and *r*, *s*∈*I*_0_, we have the following:

,
2. *x*_*t*_is (*r*,*s*)-fuzzy *θ*-cluster point of *λ*iff *x*_*t*_∈*D*_*τ*,*τ*_^∗^(*λ*,*r*,*s*).
3. *C*_*τ*,*τ*_^∗^(*λ*,*r*,*s*)≤*D*_*τ*,*τ*_^∗^(*λ*,*r*,*s*),
4. If *τ*(*λ*)≥*r*and *τ*^∗^(*λ*)≤*s*, then *C*_*τ*,*τ*_^∗^(*λ*,*r*,*s*)=*D*_*τ*,*τ*_^∗^(*λ*,*r*,*s*),
5. If *λ*is (*r*,*s*)-fpo, then *C*_*τ*,*τ*_^∗^(*λ*,*r*,*s*)=*D*_*τ*,*τ*_^∗^(*λ*,*r*,*s*),
6. If *λ*is (*r*,*s*)-fpo and *λ*=*C*_*τ*,*τ*_^∗^(*I*_*τ*,*τ*_^∗^(*λ*,*r*,*s*),*r*,*s*), then *D*_*τ*,*τ*_^∗^(*λ*,*r*,*s*)=*λ*.

The complement of (*r*,*s*)-fuzzy *θ*-closed set is called (*r*,*s*)-fuzzy *θ*-open and the (*r*,*s*)-fuzzy *θ*-interior operator denoted by *T*_*τ*,*τ*_^∗^(*λ*,*r*,*s*) is defined by .

### Remark 2.1

From Theorem 2.4 It is easy to see that:

1. *I*_*τ*,*τ*_^∗^(*λ*,*r*,*s*)≤*T*_*τ*,*τ*_^∗^(*λ*,*r*,*s*) for any *λ*∈*I*^*X*^, *r*∈*I*_0_and *s*∈*I*_1_,
2. *T*_*τ*,*τ*_^∗^(*λ*,*r*,*s*)=*I*_*τ*,*τ*_^∗^(*λ*,*r*,*s*) for each *λ*∈*I*^*X*^, *r*∈*I*_0_and *s*∈*I*_1_; *τ*(*λ*)≥*r*and *τ*^∗^(*λ*)≤*s*.

## Double Fuzzy weakly preopen functions

### Definition 3.7

A function  is said to be double fuzzy weakly preopen if 

for each *λ*∈*I*^*X*^, *r*∈*I*_0_ and *s*∈*I*_1_; *τ*_1_(*λ*)≥*r* and .

### Remark 3.2

Every double fuzzy weakly open function is double fuzzy preopen and every double fuzzy preopen function is double fuzzy weakly preopen, but the converse need not be true in general.

### Example 3.1

Let *X*={*a*,*b*,*c*} and *Y*={*x*,*y*,*z*}. Fuzzy sets *λ*_1_, *λ*_2_ and *λ*_3_ are defined as: 

Define *τ*_1_and *τ*_2_ as follows: 



Then the mapping  defined by *f*(*a*)=*z*, *f*(*b*)=*x* and *f*(*c*)=*y* is double fuzzy weakly preopen but not double fuzzy preopen. Where ,  and *f*(*λ*) is not -fpo.

### Example 3.2

Let *X*={*a*,*b*,*c*} and *Y*={*x*,*y*,*z*}. Fuzzy sets *λ*_1_, *λ*_2_ and *λ*_3_ are defined as: 

Let  and  defined as follows: 



Then the mapping  defined by *f*(*a*)=*z*, *f*(*b*)=*x*and *f*(*c*)=*y*is double fuzzy weakly preopen but not double fuzzy weakly open. Since .

### Theorem 3.5

For a function , . The following statements are equivalent:

1. *f* is double fuzzy weakly preopen,
for each *λ*∈*I*^*X*^, *r*∈*I*_0_and *s*∈*I*_1_,
for each *ν*∈*I*^*Y*^, *r*∈*I*_0_and *s*∈*I*_1_,
for each *ν*∈*I*^*Y*^, *r*∈*I*_0_and *s*∈*I*_1_.

### Proof

(1)⇒(2) Let *λ*∈*I*^*X*^ and . Then there exists  such that . Thus  and hence 

Since *f* is double fuzzy weakly preopen, 

and hence . This shows that . Thus (*f*(*λ*),*r*,*s*)) and so, .

(2)⇒(1) Let *μ*∈*I*^*X*^; *τ*_1_(*μ*)≥*r* and . Since , then 

Hence *f* is double fuzzy weakly preopen.

(2)⇒(3) Let *ν*∈*I*^*Y*^. By using (2), , . Therefore, .

(3)⇒(2) Trivial.

(3)⇒(4) Let *ν*∈*I*^*Y*^. Using (3), we have 

Therefore, we obtain , *r*,*s*).

(4)⇒(3) Similarly we obtain, , for every *ν*∈*I*^*Y*^, *r*∈*I*_0_ and *s*∈*I*_1_, i.e., . □

### Theorem 3.6

For the function . The following statements are equivalent:

1. *f* is double fuzzy weakly preopen,
2. For each *x*_*t*_ ∈ **P**(*X*) and each *μ*∈*I*^*X*^; *τ*_1_(*μ*)≥*r*and  with *x*_*t*_≤*μ*, there exists (*r*,*s*)-fpo set *γ*such that *f*(*x*_*t*_)≤*γ*and .

### Proof

(1)⇒(2) Let *x*_*t*_ ∈ **P**(*X*) and *μ*∈*I*^*X*^such that *τ*_1_(*μ*)≥*r*,  and *x*_*t*_≤*μ*. Since *f* is double fuzzy weakly preopen, then . Let . Hence (*μ*,*r*,*s*)), with *f*(*x*_*t*_)≤*γ*.

(2)⇒(1) Let *μ*∈*I*^*X*^; *τ*_1_(*μ*)≥*r*,  and *y*_*s*_≤*f*(*μ*). It follows from (2) that  for some (*r*,*s*)-fpo *γ*∈*I*^*Y*^and *y*_*s*_≤*γ*. Hence we have, . This shows that , i.e. *f* is double fuzzy weakly preopen function. □

### Theorem 3.7

Let  be a bijective function. Then the following statements are equivalent:

1. *f* is double fuzzy weakly preopen;
for each *λ*∈*I*^*X*^, *r*∈*I*_0_and *s*∈*I*_1_; *τ*_1_(*λ*)≥*r*and ;
for each *ν*∈*I*^*X*^, *r*∈*I*_0_and *s*∈*I*_1_;  and .

### Proof

(1)⇒(2) Let *ν*∈*I*^*X*^; *τ*_1_(*ν*)≥*r* and . Then we have, 

and so . Hence .

(2)⇒(3) Let *λ*∈*I*^*X*^; *τ*_1_(*λ*)≥*r* and . Since  is (*r*,*s*)-fc set and *r*,*s*) by (3) we have .

(3)⇒(2) Trivial.

(2)⇒(1) Trivial. □

### Theorem 3.8

For a function . The following statements are equivalent:

1. *f* is double fuzzy weakly preopen;
for each *ν*∈*I*^*X*^, *r*∈*I*_0_and *s*∈*I*_1_; *τ*_1_(*ν*)≥*r*and ;
for each *λ*∈*I*^*X*^, *r*∈*I*_0_and *s*∈*I*_1_; *τ*_1_(*λ*)≥*r*and ;
, for each (*r*,*s*)-fpo set *λ*∈*I*^*X*^;
, for each (*r*,*s*)-f*α*o set *λ*∈*I*^*X*^.

### Proof

(1)⇒(2) Let *ν*∈*I*^*X*^, *r*∈*I*_0_ and *s*∈*I*_1_;  and . By (1), 

(2)⇒(3) It is clear.

(3)⇒(4) Let *λ* be (*r*,*s*)-fpo set. Hence by (3), 

(4)⇒(5) and (5)⇒(1) are clear. □

### Definition 3.8

A function  is said to be double fuzzy strongly continuous, if  for each *λ*∈*I*^*X*^, *r*∈*I*_0_and *s*∈*I*_1_.

### Theorem 3.9

If  is double fuzzy weakly preopen and double fuzzy strongly continuous function, then *f* is double fuzzy preopen.

### Proof

Let *λ*∈*I*^*X*^ such that *τ*_1_(*λ*)≥*r* and . Since *f* is double fuzzy weakly preopen 

However, since *f* is double fuzzy strongly continuous, then  and therefore *f*(*λ*) is (*r*,*s*)-fpo. □

### Definition 3.9

A function  is said to be double fuzzy contra-preclosed if *f*(*λ*) is (*r*,*s*)-fpo for each *λ*∈*I*^*X*^, *r*∈*I*_0_and *s*∈*I*_1_;  and .

### Theorem 3.10

If  is double fuzzy contra-preclosed, then *f* is double fuzzy weakly preopen function.

### Proof

Let *λ*∈*I*^*X*^; *τ*_1_(*λ*)≥*r* and . Then, we have 

□

The converse of the above theorem need not be true in general as in the following Example.

### Example 3.3

Let *X*={*a*,*b*,*c*} and *Y*={*x*,*y*,*z*}. Define fuzzy sets *λ*_1_, *λ*_2_ as follows: 

Let  and  defined as follows:



Then the function  defined as *f*(*a*)=*x*, *f*(*b*)=*y*and *f*(*c*)=*z* is double fuzzy weakly preopen but it isn’t double fuzzy contra-preclosed.

### Definition 3.10

An *I*-dfts (*X*,*τ*,*τ*^∗^) is said to be (*r*,*s*)-fuzzy regular space if for each *λ*∈*I*^*X*^; *τ*(*λ*)≥*r*and *τ*^∗^(*λ*)≤*s*is a union of (*r*,*s*)-fo sets *μ*_*i*_∈*I*^*X*^such that *C*_*τ*,*τ*_^∗^(*μ*_*i*_,*r*,*s*)≤*λ*for each *i*∈*J*.

### Theorem 3.11

Let (*X*,*τ*,*τ*^∗^) be (*r*,*s*)-regular fuzzy topological space. Then,  is double fuzzy weakly preopen if and only if *f* is double fuzzy preopen.

### Proof

The sufficiency is clear. For the necessity, let *λ*∈*I*^*X*^, *r*∈*I*_0_, *s*∈*I*_1_; , *τ*_1_(*λ*)≥*r* and . For each *x*_*t*_≤*λ*, let . Hence we obtain that  and, 

Thus *f* is double fuzzy preopen. □

### Theorem 3.12

If  is double fuzzy almost open function, then it is double fuzzy weakly preopen.

### Proof

Let *λ*∈*I*^*X*^; *τ*_1_(*λ*)≥*r* and *τ*1∗(*λ*)≤*s*. Since *f* is double fuzzy almost open and  is (*r*,*s*)-fro, then 

and hence 

This shows that *f* is double fuzzy weakly preopen. □

### Definition 3.11

Let (*X*,*τ*,*τ*^∗^) be an *I*-dfts, *r*∈*I*_0_and *s*∈*I*_1_. The two fuzzy sets *λ*, *μ*∈*I*^*X*^are said to be (*r*,*s*)-fuzzy separated iff  and . A fuzzy set which cannot be expressed as a union of two (*r*,*s*)-fuzzy separated sets is said to be (*r*,*s*)-fuzzy connected.

### Definition 3.12

Let (*X*,*τ*,*τ*^∗^) an *I*-dfts. The fuzzy sets *λ*, *μ*∈*I*^*X*^such that , , are said to be fuzzy (*r*,*s*)-pre-separated if  and  or equivalently if there exist two (*r*,*s*)-fpo sets *ν*, *γ*such that *λ*≤*ν*, *μ*≤*γ*,  and . An *I*-dfts which can not be expressed as a union of two fuzzy (*r*,*s*)-pre-separated sets is said to be fuzzy (*r*,*s*)-pre-connected space.

### Theorem 3.13

If  is an injective double fuzzy weakly preopen and strongly double fuzzy continuous function from the space  onto an (*r*,*s*)-fuzzy pre-connected space , then  is (*r*,*s*)-fuzzy connected.

### Proof

Let  be not (*r*,*s*)-fuzzy connected. Then there exist (*r*,*s*)-fuzzy separated sets *β*, *γ*∈*I*^*X*^ such that . Since *β*and *γ* are (*r*,*s*)-fuzzy separated, there exists *λ*, *μ*∈*I*^*X*^; *τ*_1_(*λ*)≥*r*, *τ*_1_(*μ*)≥*r* and ,  such that *β*≤*λ*, *γ*≤*μ*,  and . Hence we have *f*(*β*)≤*f*(*λ*), *f*(*γ*)≤*f*(*μ*),  and . Since *f* is double fuzzy weakly preopen and double fuzzy strongly continuous function, from Theorem 3.10 we have *f*(*λ*) and *f*(*μ*) are (*r*,*s*)-fpo sets. Therefore, *f*(*β*) and *f*(*γ*) are (*r*,*s*)-fuzzy pre-separated and 

which is contradiction with  is (*r*,*s*)-fuzzy pre-connected. Thus  is (*r*,*s*)-fuzzy connected. □

## Double Fuzzy weakly preclosed functions

### Definition 4.13

A function  is said to be double fuzzy weakly preclosed function if 

for each *λ*∈*I*^*X*^, *r*∈*I*_0_ and *s*∈*I*_1_;  and .

### Remark 4.3

Clearly, every double fuzzy preclosed function is double fuzzy weakly preclosed, but the converse need not be true in general, as the next example shows.

### Example 4.4

Let *X*={*a*,*b*} and *Y*={*x*,*y*}. Fuzzy sets *λ*_1_ and *λ*_2_ are defined as: 

Let 



Then the function  defined by *f*(*a*)=*x*, *f*(*b*)=*y* is double fuzzy weakly preclosed but is not double fuzzy preclosed.

### Theorem 4.14

For a function . The following statements are equivalent.

1. *f* is double fuzzy weakly preclosed;
for each *λ*∈*I*^*X*^, *r*∈*I*_0_and *s*∈*I*_1_; *τ*_1_(*λ*)≥*r*and ;
for each *λ*∈*I*^*X*^, *r*∈*I*_0_and *s*∈*I*_1_;  and ;
for each (*r*,*s*)-fpc set *λ*∈*I*^*X*^, *r*∈*I*_0_and *s*∈*I*_1_;
for each (*r*,*s*)-f*α*c *λ*∈*I*^*X*^, *r*∈*I*_0_and *s*∈*I*_1_.

### Proof

Straightforward. □

### Theorem 4.15

For a function . The following statements are equivalent.

1. *f* is double fuzzy weakly preclosed;
for each (*r*,*s*)-fro set *λ*∈*I*^*X*^, *r*∈*I*_0_and *s*∈*I*_1_;
3. For each *ν*∈*I*^*Y*^, *μ*∈*I*^*X*^, *r*∈*I*_0_and *s*∈*I*_1_; *τ*_1_(*μ*)≥*r*and  with *f*^−1^(*ν*)≤*μ*, there exists (*r*,*s*)-fpo set *γ*∈*I*^*Y*^with *ν*≤*γ*and ;
4. For each fuzzy point *y*_*s*_ ∈ **P**(*Y*) and each *μ*∈*I*^*X*^, *r*∈*I*_0_and *s*∈*I*_1_such that *τ*_1_(*μ*)≥*r*and  with *f*^−1^(*y*_*s*_)≤*μ*, there exists (*r*,*s*)-fpo set *γ*∈*I*^*Y*^; *y*_*s*_≤*γ*and ;
for each *λ*∈*I*^*X*^, *r*∈*I*_0_and *s*∈*I*_1_;
for each *λ*∈*I*^*X*^, *r*∈*I*_0_and *s*∈*I*_1_;
for each (*r*,*s*)-fpo set *λ*∈*I*^*X*^, *r*∈*I*_0_and *s*∈*I*_1_.

### Proof

We will prove (2)⇒(3) and (1)⇒(6).

(2)⇒(3): Let *ν*∈*I*^*Y*^, *r*∈*I*_0_, *s*∈*I*_1_ and let *μ*∈*I*^*X*^; *τ*_1_(*μ*)≥*r* and  with *f*^−1^(*ν*)≤*μ*. Then  and consequently, . Since  is (*r*,*s*)-fro,  by (2). Let . Then *γ* is (*r*,*s*)-fpo with *ν*≤*γ* and 

(1)⇒(6): Let *ν*∈*I*^*Y*^, *r*∈*I*_0_ and *s*∈*I*_1_; ,  and . Since , there exists (*r*,*s*)-fpo *γ*∈*I*^*Y*^ with *y*_*s*_≤*γ* and  by (6). Therefore , so that . □

### Theorem 4.16

If  is double fuzzy weakly preclosed, then for each *y*_*s*_∈ **P**(*Y*) and each , there exists (*r*,*s*)-fpo set *γ*∈*I*^*Y*^; , such that .

### Proof

Let . Then *μ*(*x*) + *s*>1 and hence there exists *t*∈(0,1) such that *μ*(*x*)>*t*>1−*s*. Then . By Theorem 3.7-6 there exists (*r*,*s*)-fpo set *γ*∈*I*^*Y*^; *y*_*t*_≤*γ*such that . Now, *γ*(*y*)>*t*and hence *γ*(*y*)>1−*s*. Thus *γ*is (*r*,*s*)-fpo neighborhood of *y*_*s*_. □

### Definition 4.14

Let (*X*,*τ*,*τ*^∗^) be an *I*-dfts. A fuzzy set *λ*∈*I*^*X*^is called (*r*,*s*)-fuzzy pre-Q-neighborhood of *x*_*t*_if there exists (*r*,*s*)-fpo set *μ*∈*I*^*X*^such that *x*_*t*_*qμ*≤*λ*. We denote the set of all (*r*,*s*)-fuzzy pre-Q-neighborhood of *x*_*t*_by **PQ**_*τ*,*τ*_^∗^(*x*_*t*_,*r*,*s*).

### Theorem 4.17

In an *I*-dfts (*X*,*τ*,*τ*^∗^). A fuzzy point *x*_*t*_∈*PC*_*τ*,*τ*_^∗^(*λ*,*r*) if and only if for every *μ* ∈ **PQ**_*τ*,*τ*_^∗^(*x*_*t*_,*r*,*s*), *μqλ*is hold.

### Proof

Straightforward. □

### Theorem 4.18

If  is double fuzzy weakly preclosed and if for each *ν*∈*I*^*X*^, *r*∈*I*_0_and *s*∈*I*_1_; ,  and each  there exists  such that . Then *f* is double fuzzy preclosed.

### Proof

Let *ν*∈*I*^*X*^, *r*∈*I*_0_and *s*∈*I*_1_; ,  and let . Then , and hence there exists  such that . Since *f* is double fuzzy weakly preclosed by using Theorem 3.12, there exists (*r*,*s*)-fuzzy pre-Q-neighborhood *γ*∈*I*^*Y*^with *y*_*s*_≤*γ*and . Therefore, we obtain  and hence , this shows that . Therefore, *f*(*ν*) is (*r*,*s*)-fpc and *f* is double fuzzy preclosed function. □

### Definition 4.15

A function  is said to be double fuzzy contra-open (resp. double fuzzy contra-closed) if  and (resp. *τ*_2_(*f*(*λ*))≥*r*and ) for each *λ*∈*I*^*X*^, *r*∈*I*_0_and *s*∈*I*_1_; *τ*_1_(*λ*)≥*r*and  (resp.  and ).

### Theorem 4.19

If  is double fuzzy contra-open, then *f* is double fuzzy weakly preclosed.

### Proof

Let *λ*∈*I*^*X*^, *r*∈*I*_0_ and *s*∈*I*_1_ such that  and . Then, 

□

### Theorem 4.20

If  is double fuzzy weakly preclosed, then for every *ν*∈*I*^*Y*^and every *λ*∈*I*^*X*^, *r*∈*I*_0_, *s*∈*I*_1_such that *τ*_1_(*λ*)≥*r*and  with *f*^−1^(*ν*)≤*λ*, there exists (*r*,*s*)-fpc set *γ*∈*I*^*Y*^such that *ν*≤*γ*and .

### Proof

Let *ν*∈*I*^*Y*^and let *λ*∈*I*^*X*^, *r*∈*I*_0_and *s*∈*I*_1_such that *τ*_1_(*λ*)≥*r*and  with *f*^−1^(*ν*)≤*λ*. Put , then *γ*is (*r*,*s*)-fpc set in *I*^*Y*^such that *ν*≤*γ*since . And since *f* is double fuzzy weakly preclosed, . □

### Corollary 4.21

If  is double fuzzy weakly preclosed, then for every *y*_*s*_∈ **P**(*Y*) and every *λ*∈*I*^*X*^, *r*∈*I*_0_and *s*∈*I*_1_such that *τ*_1_(*λ*)≥*r*and  with *f*^−1^(*y*_*s*_)≤*λ*, there exists (*r*,*s*)-fpc set *γ*∈*I*^*Y*^; *y*_*s*_≤*γ*such that .

### Definition 4.16

A fuzzy set *λ*∈*I*^*X*^is called (*r*,*s*)-fuzzy *θ*-compact if for each family {*μ*_*i*_∣*i*∈*J*} in  satisfy  for each *x*∈*X*, there exist a finite subset *J*_0_of *J* such that .

### Theorem 4.22

If  is double fuzzy weakly preclosed with all fibers (*r*,*s*)-fuzzy *θ*-closed, then *f*(*λ*) is (*r*,*s*)-fpc for each (*r*,*s*)-fuzzy *θ*-compact *λ*∈*I*^*X*^, *r*∈*I*_0_and *s*∈*I*_1_.

### Proof

Let *λ* be (*r*,*s*)-fuzzy *θ*-compact and let . Then  and for each *x*_*t*_≤*λ* there is  with  and . Clearly  satisfy  for each *x*∈*X* and since *λ* is (*r*,*s*)-fuzzy *θ*-compact, there is  such that , where . Since *f* is double fuzzy weakly preclosed, by using Theorem 3.12 there exists  with 

Therefore *y*_*s*_≤*γ*and . Thus . Thus *f*(*λ*) is (*r*,*s*)-fpc set. □

### Definition 4.17

Let (*X*,*τ*,*τ*^∗^) be an *I*-dfts. The fuzzy sets *λ*, *μ*∈*I*^*X*^are (*r*,*s*)-fuzzy strongly separated if there exist *ν*, *γ*∈*I*^*X*^such that *τ*(*ν*)≥*r*and *τ*^∗^(*ν*)≤*s*, *τ*(*γ*)≥*r*with *λ*≤*ν*, *μ*≤*γ*and .

### Definition 4.18

An *I*-dfts (*X*,*τ*,*τ*^∗^) is called (*r*,*s*)-fuzzy pre *T*_2_if for each ,  with different supports there exists (*r*,*s*)-fpo sets *λ*, *μ*∈*I*^*X*^such that ,  and .

### Theorem 4.23

If  is double fuzzy weakly preclosed surjection and all fibers are (*r*,*s*)-fuzzy strongly separated, then  is (*r*,*s*)-fuzzy pre-*T*_2_.

### Proof

Let  and let *γ*,*ν*∈*I*^*X*^, *r*∈*I*_0_and *s*∈*I*_1_; *τ*_1_(*γ*)≥*r*, , *τ*_1_(*ν*)≥*r*and *τ*_1_(*ν*)≤*s*such that  and  respectively with . By using Theorem 3.12-4 there are (*r*,*s*)-fpo sets *λ*, *μ*∈*I*^*Y*^such that  and ,  and . Therefore , because  and *f* is surjective. Thus  is (*r*,*s*)-fuzzy pre-*T*_2_. □

### Definition 4.19

an *I*-dfts (*X*,*τ*,*τ*^∗^) is said to be (*r*,*s*)-extremally disconnected if *τ*(*C*_*τ*,*τ*_^∗^(*λ*,*r*,*s*))≥*r*and *τ*^∗^(*C*_*τ*,*τ*_^∗^(*λ*,*r*,*s*))≤*s*for each *λ*∈*I*^*X*^; *τ*(*λ*)≥*r*and *τ*^∗^(*λ*)≤*s*.

### Definition 4.20

an *I*-dfts (*X*,*τ*,*τ*^∗^) is said to be (*r*,*s*)-fuzzy almost compact if for each (*r*,*s*)-fuzzy open cover {*λ*_*i*_∣*i*∈*J*} of *X*, there is a finite subset *J*_0_of *J* such that .

### Definition 4.21

A fuzzy set *λ*in an *I*-dfts (*X*,*τ*,*τ*^∗^) is said to be (*r*,*s*)-fuzzy p-compact iff for each family of (*r*,*s*)-fpo sets {*μ*_*i*_∣*i*∈*J*} satisfies  for each *x*∈*X*. There exists finite subfamily *J*_0_of *J* such that  for each *x*∈*X*.

### Theorem 4.24

Let  be (*r*,*s*)-extremally disconnected *I*-dfts. Let  be double fuzzy open and double fuzzy preclosed injective function such that *f*^−1^(*y*_*s*_) is (*r*,*s*)-fuzzy almost compact for each *y*_*s*_ ∈ **P**(*Y*). If *λ*∈*I*^*Y*^is (*r*,*s*)-fuzzy P-compact. Then *f*^−1^(*λ*) is (*r*,*s*)-fuzzy almost compact.

### Proof

Let {*ν*_*j*_∣æ∈*J*} be (*r*,*s*)-fuzzy open cover of *f*^−1^(*λ*). Then for each *y*_*s*_≤*λ*∧*f*(*X*), , for some finite subfamily *J*(*y*_*s*_) of *J*. Since  is (*r*,*s*)-extremally disconnected each  and , hence  and . So by Corollary 4.21 there exists (*r*,*s*)-fpc set  such that . Then,  is (*r*,*s*)-fuzzy preclosed cover of *λ*,  for some finite fuzzy subset *K* of *λ*∧*f*(*X*). Hence, 

so . Therefore *f*^−1^ (*λ*) is (*r*,*s*)-fuzzy almost compact. □
